# Foreign Correspondence

**Published:** 1869-10

**Authors:** 


### Foreign Correspondence.

HEIDELBERG, *August 13, 1869.*

DEAR EXAMINER:—Being in Zurich a few days ago, I took occasion to visit the Canton's Hospital, which has about four hundred beds. I first attended the clinic of Prof. Horner, who has charge of the department of the eye, and saw some operations for cataract and the removal of pterygii. Prof H., though a comparatively young man, is a careful operator and instructive lecturer. Like most other operators on the eye whom I have seen in Austria and Germany, he ordinarily operates without an anæsthetic. What the comparative successes of eye operations with and without chloroform are, I am unable to say. I am not aware that there are statistics on the point; nevertheless, I have been led to believe from witnessing numerous operations, that if there were any difference in results, the more favorable they must be with the use of chloroform. I have seen many patients who were entirely powerless to hold the eye fixed on the approximation of the knife, consequently its point has had its entrance and exit far from the place intended, and once, through a sudden movement of the eye, came out through the centre of the cornea; and for the same reasons the iris and other structures of the eye are often mutilated, causing iritis, keratitis, etc., and often loss of the organ, apparently, at least, through unnecessary irritation, which in a great degree might have been avoided with chloroform.

I also noticed that Prof. Horner did not release his hold on the conjunctiva with the pincettes, or remove the speculum until the operation was entire, while others have told me that this mode of procedure was more or less dangerous, as the patient often brings so much pressure to bear as to rupture the capsule of the lens and cause the escape of more or less of the vitreous body, which does not so often happen if the pincettes are removed before the flap is completed. Although this is not an eye journal, Mr. Editor, yet as the general profession is beginning more and more to look up these departments called specialties, I have inserted these observations as perhaps interesting questions. Prof. H. has about thirty eye patients in his wards, and five to twelve out patients, that visit his clinic at the hospital. His assistant gives a course on ophthalmoscopy from time to time, but the material is not very abundant. The number of eye students is also small.

I next visited the surgical clinic of Prof. Rose, who had many interesting cases. Prof. Rose is successor to Prof. Bilroth, of Vienna, and though a man of not so extensive and profound learning as Prof. B., yet is quite efficient for his position, and fills it with satisfaction to his students. The surgical class is small—perhaps thirty or more—and enjoys the best of advantages for witnessing operations, and its members are often permitted to perform many important ones. I made the morning visit with the Professor, and saw about eighty instructive cases. I was convinced that Zurich is a better place for surgery than Vienna. I think Prof. Billroth has confessed the fact since he went to Vienna. In Vienna, one sees a large number of ulcers, necroses, strumous tumors, etc., while in Zurich, one sees a larger number of fractures, amputations, and wounds. I saw a child, nine months old, that had prolapsus ani for six months. All forms of treatment had been fruitless. The Professor was using no carbolic acid in any of his cases, but preparations of zinc, potash, etc. Prof. Biermer gives a medical clinic five times a week. Prof. Gusserow gives an obstetric and gynäkologische clinic with touchirübungen four times a week; also, an operative midwife course three times a week. The number of births in the hospital, however, scarcely exceed two hundred a year, consequently this branch is practically better studied elsewhere.

I saw several female students from America, England, and Russia, at the clinics, who, on account of the moral salubrity of the city and the respect shown them by the students, find it an agreeable place to study. The whole number of medical students, I think, is less than a hundred.

In Lerne, I had the pleasure of meeting Prof. Jewell, who for several semesters has ably filled the chair of anatomy in Chicago Medical College, and is now on a visit to this country and the East, for studies in philosophy, biblical history, etc. The Professor has already achieved a fair fame as a lecturer and writer on science and metaphysics, and is rapidly widening his reputation. May success attend him in his new field of labor.

The Heidelberg University has for generations been one of the most celebrated of Europe, and in many respects is still entitled to the appellation; yet larger hospitals have attracted students elsewhere, so that of the eight hundred or more students who resort here for learning, barely more than a hundred are students of medicine. The city has only about 17,000 inhabitants—hence its hospital wards cannot be very rich in material. There are, however, in the general hospital about one hundred and forty patients, and in the eye hospital thirty-five. Surgical students enjoy very good advantages here, as the number is small, and Prof. Simon allows them to perform nearly all the operations, as amputations, resections, etc. Prof. Moos gives courses on the ear of 6–8 weeks duration, and has a tolerable supply of patients. Prof. Becker has an ophthalmic clinic four times a week at the eye hospital, and has about six out patients daily. He also gives instruction in the use of the ophthalmoscope three times a week, for which patients come from the blind institute, in an adjacent town. The class also visit Mannheim, a city half an hour by rail distant, twice a week, to see cases. Above all, however, Heidelberg is to be recommended to the student of anatomy, chemistry, and physiology, for which he can enjoy ten-fold better opportunities than in Vienna, or many other larger schools. Profs. Bunsen, Helmholtz, Arnold, Nuhn, etc., who occupy these chairs, attract justly many admirers. Prof. Wundt gives a course of lectures on psychology, also on anthropology. Prof. Helmholz gives a course on the "general results of science," with especial reference to biology. I read awhile ago that Prof. Bunsen had lost his second eye by an explosion. Such is not the case. I am told he was but slightly injured, and has now entirely recovered.

Yours, truly, F.

